# Naturally occurring polymorphisms in the virulence regulator Rsp modulate *Staphylococcus aureus* survival in blood and antibiotic susceptibility

**DOI:** 10.1099/mic.0.000695

**Published:** 2018-07-20

**Authors:** Aishwarya Krishna, Matthew T. G. Holden, Sharon J. Peacock, Andrew M. Edwards, Sivaramesh Wigneshweraraj

**Affiliations:** ^1^​ MRC Centre for Molecular Bacteriology and Infection, Imperial College London, London, UK; ^2^​ Wellcome Trust Sanger Institute, Hinxton, UK; ^3^​ School of Medicine, University of St Andrews, St Andrews, UK; ^4^​ London School of Hygiene and Tropical Medicine, London, UK

**Keywords:** *Staphylococcus aureus*, Rsp, virulence regulator, mutations, bacteraemia

## Abstract

Nasal colonization by the pathogen *Staphylococcus aureus* is a risk factor for subsequent infection. Loss of function mutations in the gene encoding the virulence regulator Rsp are associated with the transition of *S. aureus* from a colonizing isolate to one that causes bacteraemia. Here, we report the identification of several novel activity-altering mutations in *rsp* detected in clinical isolates, including for the first time, mutations that enhance *agr* operon activity. We assessed how these mutations affected infection-relevant phenotypes and found loss and enhancement of function mutations to have contrasting effects on *S. aureus* survival in blood and antibiotic susceptibility. These findings add to the growing body of evidence that suggests *S. aureus* ‘trades off’ virulence for the acquisition of traits that benefit survival in the host, and indicates that infection severity and treatment options can be significantly affected by mutations in the virulence regulator *rsp*.

## Full-Text

The Gram-positive bacterium *Staphylococcus aureus* is a major pathogen that causes a range of human infections [1]. An effective immune response supports the rapid resolution of superficial skin and soft tissue infections caused by *S. aureus,* often without the need for treatment. However, entry into the bloodstream can lead to the metastatic spread of *S. aureus* and the establishment of more severe infections at distal sites, some of which can be difficult to treat [1, 2].

Although capable of causing infections, *S. aureus* exists primarily as a commensal organism, colonizing the anterior nares of an estimated ~30 % of the population. Nasal carriage is a significant risk factor for infection [3–5], and several genetic analyses have confirmed that infecting strains are typically those colonizing the individual, with only a few genetic alterations [6–9]. One longitudinal study identified a protein-truncating mutation in an AraC-type transcription regulator Rsp (repressor of surface proteins) that preceded, and was implicated in the development of, *S. aureus* bacteraemia [7]. Subsequently, a single mutation resulting in the A204P substitution in the AraC DNA-binding domain of Rsp was found to be the only difference between carriage and bacteraemia isolates from another infected patient [8, 9].

Rsp is a highly conserved DNA-binding regulator of the accessory gene regulator (*agr*) operon in *S. aureus* that regulates the expression of nearly all virulence factors in a cell density-dependent manner [10, 11]. Despite this important role in virulence factor expression, *agr* dysfunctional strains are often isolated from bloodstream infections, and have been associated with increased duration of and mortality attributed to *S. aureus* bacteraemia [12–18]. Given that Rsp is a regulator of the *agr* operon [10], and loss of function mutations in *rsp* have previously been associated with *S. aureus* bacteraemia [7, 9], we aimed to investigate the phenotypic consequences and clinical implications of naturally occurring *rsp* mutations in methicillin-resistant *S. aureus* (MRSA). We interrogated 1429 published whole genome sequences of clinical isolates belonging to sequence type 22 (ST22) [19–24], a globally successful MRSA clone, for mutations in *rsp* as previously described [23]. To exclude intra-species polymorphisms in *rsp* from our analysis, *rsp* from the ST22 MRSA reference sequence (strain HO 5906 0412) [23] was compared to all complete *S. aureus* genomes (212) available on the NCBI Genome database. This revealed a high level of sequence conservation (≥99 % identity) and validated the identified non-synonymous mutations.

**Fig. 1.**
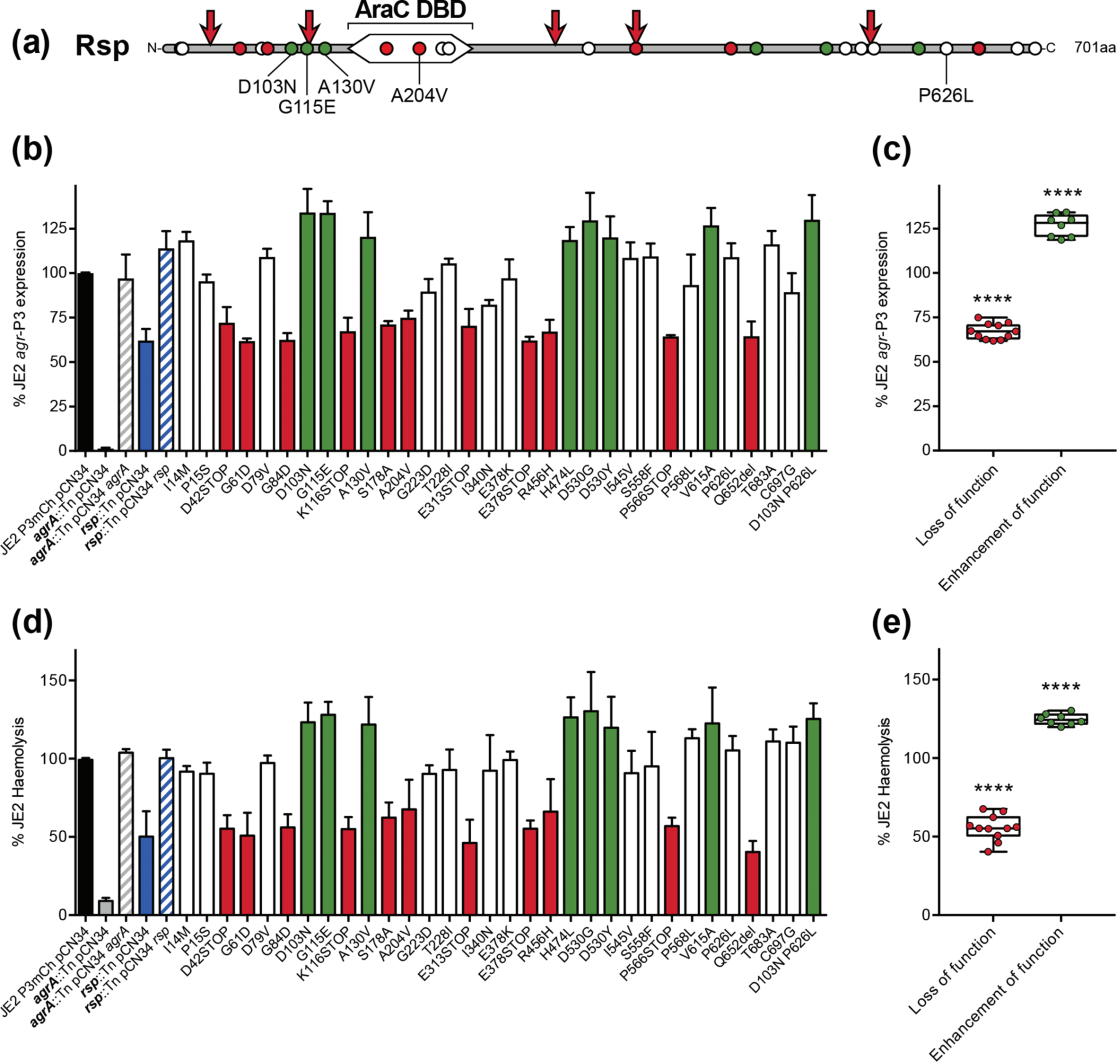
Identification and investigation of the effect of *rsp* mutations on *agr* expression and haemolysin production. (a) Schematic representation of Rsp with the AraC DNA-binding domain (DBD) indicated. Arrows show sites of truncation events while points represent individual amino acid substitutions, coloured red, green or white to indicate ‘loss of function’, ‘enhancement of function’ and non-altering mutations in *rsp*, respectively. Growth-normalized *agr* operon activity (b) and haemolysin production (d) of strains at the 16 h time point are expressed relative to the wild-type reporter strain JE2 P3mCh pCN34. Loss of function, enhancement of function and non-altering mutations are coloured red, green and white, respectively. For brevity, transposon disruption by *bursa aurealis* is noted as '::Tn’. *agr* operon expression (c) and haemolysin production (e) relative to the wild-type reporter strain of loss of function and enhancement of function *rsp* mutations. Each point represents the mean of three independent experiments conducted for each *rsp* mutant. The median is represented by the horizontal line, with box and whiskers showing the interquartile range and range. *P*-values were obtained by one-way ANOVA compared to the wild-type with Dunnett’s post hoc correction (**** *P*≤0.0001).

Twenty-seven novel substitutions and five truncation mutations in *rsp* were identified in 39 clinical isolates (Fig. 1a). The most common mutation, present in four geographically distinct clinical isolates, resulted in the D103N amino acid substitution, preceding the DNA-binding domain. This substitution was also found in combination with P626L in an additional isolate. The AraC DNA-binding domain of Rsp was the site of four mutations including one that resulted in the A204V substitution, similar to A204P reported previously [8, 9]. To assess the impact of these mutations on Rsp function, namely the ability to activate *agr* operon expression, an integrated *agr-*P3-mCherry promoter fusion construct (P3mCh) was employed and mCherry fluorescence quantified relative to optical density at 600 nm over a 16 h period [25]. The 32 identified *rsp* mutations were introduced into the wild-type pCN34 *rsp* complementation vector via site-directed mutagenesis, and wild-type and mutant plasmids were transformed into the MRSA USA300 strain JE2 carrying the *bursa aurealis* transposon in *rsp* (*rsp *:: Tn) [26], following a published protocol [27]. Sequence analysis revealed that Rsp from the ST22 reference sequence and MRSA USA300 JE2 differed at only a single amino acid, residue 75. In ST22 *S. aureus* HO 5906 0412 residue 75 is a phenylalanine, while in JE2 a tyrosine. As these amino acids differ by only a hydroxyl group [28], we deemed this mutation unlikely to have a significant effect on Rsp function. The presence of the *bursa aurealis* transposon rendered *S. aureus* strains erythromycin resistant and hence were grown in tryptic soy broth (TSB) supplemented with 10 µg erythromycin ml^−1^ [26]. Maintenance of pCN34 was achieved via the addition of 90 µg kanamycin ml^−1^ [29]. Transposon mutants of *rsp* (*rsp *:: Tn) and the master regulator of the *agr* operon *agrA* (*agrA *:: Tn) [26], along with their respective pCN34-complemented strains [30], performed as experimental controls to validate our findings.

As shown in Fig. 1(b), expression from *agr*-P3 of the four control strains and 32 *rsp* mutants was compared to the wild-type reporter strain JE2 P3mCh carrying empty pCN34 (JE2 P3mCh pCN34) after 16 h of growth in TSB at 37 °C, when *agr*-P3 expression plateaued. As expected, disruption of *agrA*, the master regulator of the *agr* operon by transposon insertion, led to a 99 % reduction in *agr* P3 expression, which returned to wild-type levels upon complementation [30]. Similarly, the *rsp* :: Tn strain showed a 38 % reduction in *agr*-P3 expression, which was restored by complementation. Eleven of the 32 *rsp* mutants exhibited a significant reduction in *agr*-P3 expression, ≤75 % of the wild-type strain (Fig. 1c). These mutations were therefore referred to as ‘loss of function’ and included the five truncation mutations in Rsp and the A204V substitution. Mutation of residue 204 in the AraC DNA-binding domain from alanine to proline has previously been predicted to abrogate Rsp function [8, 9], further validating our findings. In addition, eight ‘enhancement of function’ mutations were found to significantly increase *agr*-P3 expression by ≥120 % of wild-type (Fig. 1c), including that conferring the most common substitution in the collection, D103N. The elevated *agr*-P3 levels exhibited by the double-substitution D103N/P626L were attributed to the presence of the D103N substitution, as P626L *per se* did not affect *agr*-P3 expression (Fig. 1b).

To confirm that the observed alterations in *agr*-P3 expression were directly affecting *agr*-dependent target gene expression, *agr*-dependent haemolysin production was quantified as described previously [31]. Disruption of *agrA* and *rsp* by transposon insertion resulted in 92 and 50 % reductions in haemolysin production compared to the wild-type strain, respectively (Fig. 1d). Haemolysin production by both strains was returned to wild-type levels upon complementation. Activity-altering mutations in *rsp* that reduced or enhanced *agr*-P3 expression correspondingly affected haemolysin production (Fig. 1d, e), thus phenotypically validating the results obtained via the *agr-*P3-mCherry promoter fusion construct.

**Fig. 2.**
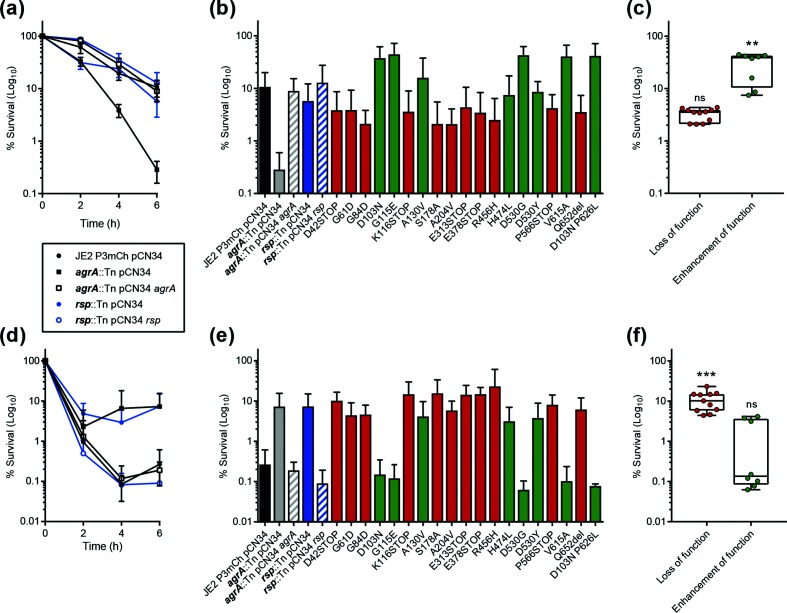
Effect of activity-altering *rsp* mutations on *S. aureus* survival in whole human blood (top) and in the presence of the lipopeptide antibiotic daptomycin (bottom). Percentage survival of strains in blood (a) and in a supra-MIC concentration of daptomycin (d) over 6 h is expressed relative to total inoculum at time 0. Percentage survival of *rsp* mutants following 6 h incubation in blood (b) or daptomycin (e). Loss of function and enhancement of function mutations are coloured red and green, respectively. For brevity, transposon disruption by *bursa aurealis* is noted as '::Tn’. Percentage survival of loss of function and enhancement of function *rsp* mutations in blood (c) and daptomycin (f). Each point represents the mean of four independent experiments conducted for each *rsp* mutant. The median is represented by the horizontal line, with box and whiskers showing the interquartile range and range. *P*-values were obtained by one-way ANOVA compared to the wild-type with Dunnett’s post hoc correction (*** *P*≤0.001, ** *P*≤0.01).

Dysfunction of both *agr* and *rsp* have been associated with *S. aureus* bacteraemia [7, 13, 16–18]. Therefore, an *ex vivo* whole human blood model was employed to assess the effect of activity-altering mutations in *rsp* on *S. aureus* survival and resistance to killing by immune components. Assays were performed as described previously [32], using freshly drawn whole blood from four independent healthy human donors. In line with previous findings [32], disruption of the master regulator of the *agr* operon, *agrA*, led to a >tenfold decrease in *S. aureus* survival compared to the wild-type strain following 6 h incubation in whole human blood (Fig. 2a, b). Although transposon disruption of *rsp* resulted in a reduction in *agr* expression (Fig. 1), no corresponding decrease in *S. aureus* survival was observed (Fig. 2a, b). Similarly, as previously reported for the truncation and A204P substitution mutants of Rsp [9], loss of function mutations in *rsp* did not significantly alter *S. aureus* survival in blood (Fig. 2c). Conversely, *rsp* mutations that enhanced *agr* operon expression (Fig. 1c) were found to significantly improve *S. aureus* survival following 6 h incubation in blood (Fig. 2c).

The findings of the *ex vivo* whole human blood model are in support of published accounts that suggest *agr-*regulated virulence factors involved in immune evasion and host cell destruction are required for *S. aureus* survival in human blood [32, 33]. Moreover, the results reported here indicate that there is a requirement for a basal level of *agr* expression for *S. aureus* survival in the bloodstream. We hypothesize that below this level, *agr*-dependent immune evasion and virulence factors are not able to effectively defend *S. aureus* against host immune attack [34]. While the identification of *rsp* mutants that increased both *agr*-P3 expression (Fig. 1b, c) and *S. aureus* survival in whole human blood at 6 h (Fig. 2b, c) suggests that an increase in *agr* expression favours survival in blood, we cannot discount the possibility that these *rsp* mutants affect the expression of *agr*-independent factors that may influence *S. aureus* survival in the bloodstream.

In addition to killing by the host immune system, antibiotics present an additional threat to *S. aureus* in the bloodstream [2, 35]. Increased tolerance to vancomycin and daptomycin, two first-line therapies for MRSA bacteraemia, has been associated with dysfunction of the *agr* operon [36–41]. We therefore determined whether loss and/or enhancement of function mutations in *rsp* affected the minimal inhibitory concentration (MIC) of vancomycin and/or daptomycin using the broth microdilution approach [42]. As shown in Table 1, the wild-type strain JE2 P3mCh pCN34 exhibited MICs for vancomycin and daptomycin in line with published reports [41, 42]. Despite an association between *agr* dysfunction and an increased tolerance to vancomycin being widely reported [36–40], disruption of *agrA* by transposon insertion did not alter the vancomycin MIC, as reported previously [41]. A twofold increase in the daptomycin MIC was, however, observed for the *agrA* transposon mutant, which was returned to wild-type levels upon complementation. Similarly, disruption of *rsp* by transposon insertion or loss of function mutation led to a twofold, complementable increase in the MIC of both first-line antibiotics. Mutations in *rsp* that enhanced *agr* operon expression showed an inconsistent effect on vancomycin MIC, but did not affect daptomycin MIC.

**Table 1.** Effect of activity-altering *rsp* mutations on the minimal inhibitory concentration of vancomycin and daptomycin Data represent the median value of three independent experiments.

	MIC (μg ml^−1^)	
	Vancomycin	Daptomycin
JE2 P3mCh pCN34	1.0	0.5
*agrA *:: Tn pCN34	1.0	1.0
*agrA *:: Tn pCN34 *agrA*	1.0	0.5
*rsp *:: Tn pCN34	2.0	1.0
*rsp *:: Tn pCN34 *rsp*	1.0	0.5
Loss of function mutation		
D42STOP	2.0	1.0
G61D	2.0	1.0
G84D	2.0	1.0
K116STOP	2.0	1.0
S178A	2.0	1.0
A204V	2.0	1.0
E313STOP	2.0	1.0
E378STOP	2.0	1.0
R456H	2.0	1.0
P566STOP	2.0	1.0
Q652del	2.0	1.0
Enhancement of function mutation		
D103N	1.0	0.5
G115E	1.0	0.5
A130V	2.0	1.0
H474L	2.0	1.0
D530G	1.0	0.5
D530Y	2.0	1.0
V615A	2.0	0.5
D103N P626L	2.0	0.5

As the increase in vancomycin MIC displayed by the *rsp* transposon mutant and loss of function mutants is unlikely to be due to the corresponding reduction in *agr* expression, we hypothesize that it may be due to alterations in cell wall physiology. A thickening of the *S. aureus* cell wall has previously been associated with an increased tolerance to vancomycin [43–45], and Rsp has been shown to regulate the expression of several genes involved in cell wall homeostasis [10]. We therefore hypothesize that Rsp regulates cell wall physiology in an *agr*-independent manner, and thus disruption and loss of function mutation in *rsp* increases tolerance to vancomycin.

The effect of *rsp* mutation on the killing kinetics of *S. aureus* by daptomycin was investigated in greater detail via a 6 h killing assay performed in Mueller–Hinton Broth (MHB) supplemented with Ca^2+^ (50 µg ml^−1^) and Mg^2+^ (10 µg ml^−1^) [41, 42]. Daptomycin was used at a concentration of 5 µg ml^−1^, 10x the wild-type MIC (Table 1). Disruption of *agrA* led to an initial period of killing by daptomycin, with percentage survival comparable to the wild-type strain at the 2 h time point. This was followed by recovery to tenfold greater survival than wild-type at the 6 h time point (Fig. 2d, e), in line with previous findings [41]. The initial period of killing followed by recovery phenotype was also exhibited by the *rsp* transposon mutant, that recovered >tenfold than the wild-type strain at 6 h of incubation with daptomycin (Fig. 2d, e). Similarly, loss of function mutations in *rsp* were found to significantly enhance survival in daptomycin, while enhancement of function mutations were unable to do so (Fig. 2f).

Supported by previous findings [41], we hypothesize that loss of function mutations in *rsp* reduce *agr* operon expression and corresponding *agr*-dependent phenol-soluble modulin α (PSMα) production. This favours the sequestration of daptomycin in released phospholipid, preventing it targeting the cell membrane and inducing cell death. Conversely, enhancement of function *rsp* mutations cause an overexpression of *agr* and PSMα that compete for released phospholipid, preventing sequestration of daptomycin for protection.

The virulence regulator Rsp and its substitutions have been the focus of several recent publications [8–10, 46]. In this study, we build on this growing body of work by identifying multiple novel mutations in MRSA clinical isolates, including several ‘enhancement of function’ mutations in *rsp* that significantly increased expression of the major virulence regulator *agr* (Fig. 1c). Three such mutations were found to precede the AraC DNA-binding domain, resulting in the D103N, G115E and A103V substitutions (Fig. 1a). Although unlikely to directly affect Rsp's interaction with RNA polymerase and DNA-binding ability – both functions performed by the AraC DNA-binding domain [10, 47] – these substitutions may alter Rsp’s interaction with other transcription regulatory factors. For example, an AraC Negative Regulator (ANR) was recently found to bind to the region preceding the AraC DNA-binding domain of AggR, preventing DNA binding [48, 49]. Therefore, it is plausible that D103N, G115E and A130V enhancement of function substitutions in Rsp may prevent binding of possible ANRs, ultimately promoting *agr*-P3 expression.

The effect of novel activity-altering mutations in *rsp* on clinically relevant phenotypes was investigated further, with emphasis on phenotypes relevant to *S. aureus* bacteraemia – survival in whole human blood and in first-line antibiotics used in the treatment of MRSA bacteraemia. We discovered that enhancement of function mutations in *rsp*, while promoting expression of *agr*-P3 (Fig. 1c) and survival in whole human blood (Fig. 2c), did not affect the susceptibility of *S. aureus* to vancomycin or daptomycin (Table 1 and Fig. 2f). Conversely, loss of function mutations in *rsp*, while reducing *agr*-P3 expression (Fig. 1c), did not affect *S. aureus* survival in whole human blood (Fig. 2c) but enhanced survival at a supra-MIC concentration of daptomycin (Fig. 2f). These findings led us to propose that via a mutation of *rsp*, *S. aureus* can modulate or ‘trade off’ the production of traditional virulence determinants with contradicting consequences for antibiotic tolerance and its ability to survive immune attack and cause disease.

The notion that *S. aureus* compromises or ‘trades off’ its production of virulence determinants to acquire additional traits is relatively new, and is in direct conflict with the traditional paradigm of loss of *agr* reducing *S. aureus* virulence [46, 50]. Low-toxicity *S. aureus* isolates, including mutants of *agr* and *rsp*, were recently shown to have enhanced fitness in the presence of human serum compared to high-toxic isolates [50]. In combination with mathematical modelling, it was proposed that a high-toxicity state facilitates the spread and transmission of *S. aureus* between hosts, while low toxicity promotes in-host survival. The results of our study are in line with and augment these findings, leading us to hypothesize that the low-toxicity state established via loss of function mutation of *rsp* would lead to more invasive infections that are also more difficult to treat with current therapeutics. This ‘trade-off’ may thus explain the frequent isolation of *agr* dysfunctional strains from invasive *S. aureus* bacteraemia infections [12–16, 18], and the acquisition of mutations in *rsp* leading to *S. aureus* bacteraemia [7–9].

In summary, the findings of this study, in addition to others recently published [9, 50], demonstrate that mutations within *rsp* can have significant impacts on staphylococcal toxin production and bacterial survival against the twin threats of host defences and antibiotics.
